# Bridging spiking neuron models and mesoscopic population models - a general theory for neural population dynamics

**DOI:** 10.1186/1471-2202-16-S1-P79

**Published:** 2015-12-18

**Authors:** Tilo Schwalger, Moritz Deger, Wulfram Gerstner

**Affiliations:** 1 Brain Mind Institute, École Polytechnique Fédérale de Lausanne (EPFL) Station 15, 1015 Lausanne, Switzerland

## 

Many circuits of the brain can be described by a system of interacting neural populations that are approximately homogeneous. For instance, cortical layers typically consist of a few main types of excitatory and inhibitory neurons that form small homogeneous populations of neurons. Such systems can be modeled on different spatial scales. On the microscopic scale, single cell activity has been faithfully described by reduced phenomenological neuron models [1]. Simulations of networks of such neuron models are, however, computationally expensive and do not offer much analytical insight. On the other hand, mesoscopic population models are reduced descriptions of the global activities of each population. These activities are stochastic due to the finite sizes of the populations. Mesoscopic models can be efficiently simulated and provide a better understanding of the dynamics owing to the abstraction of microscopic information. However, it is largely unknown how to relate mesoscopic population models to microscopic properties such as neural refractoriness, synaptic conductance dynamics and spike-frequency adaptation.

**Figure 1:**
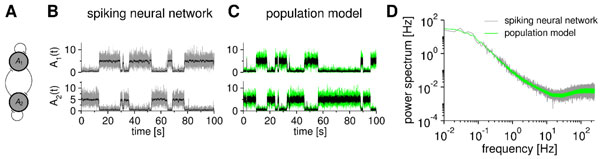
**Stochastic population equation precisely captures the collective bistable dynamics of a spiking neural network**. **A **Two mutually inhibitory populations of 500 neurons each. **B,C **Sample paths of the spiking neural network and the derived population model, respectively. Transitions are due to finite-size fluctuations. **D **The population activity of microscopic and mesoscopic model have the same first- and second-order statistics.

Here, we derive a mesoscopic population model for microscopic networks of generalized integrate-and-fire neurons [1]. This type of neuron model supports important properties like neural refractoriness, multiple-time-scale adaptation, stochastic spike generation and synaptic dynamics; its parameters can be directly extracted from experiments of real neurons. In particular, we use a mean-field and a quasi-renewal approximation [1] to derive stochastic integral equations for the population rates. These equations highlight how the history of activities and fluctuations affects the refractoriness of the populations and the activities at the current time. They can be solved forward in time and thus allow to quickly generate stochastic samples of spontaneous or evoked activities (Fig. 1B,C) that have the same statistics as a corresponding microscopic network simulation to a high degree of accuracy (Fig. 1D). The theory not only captures linear population dynamics [2] but also nonlinear collective effects that emerge on the population level such as metastability (Fig. 1). Our novel theory establishes a general framework for modeling neural population dynamics based on microscopic neuronal parameters. It offers an efficient way to analyze cortical circuits and its computational functions, and how they depend on single-cell and synaptic properties.
